# An observational field study of porcine post-weaning diarrhea: clinical and microbiological findings, and fecal pH-measurements as a potential diagnostic tool

**DOI:** 10.1186/s40813-023-00325-x

**Published:** 2023-07-11

**Authors:** Esben Østergaard Eriksen, Egle Kudirkiene, Kristiane Barington, Nicole Bakkegård Goecke, Sophie Amalie Blirup-Plum, Jens Peter Nielsen, John Elmerdahl Olsen, Henrik Elvang Jensen, Karen Pankoke, Lars Erik Larsen, Gang Liu, Ken Steen Pedersen

**Affiliations:** 1 Faculty of Health and Medical Sciences, Department of Veterinary and Animal Sciences, University of Copenhagen, Grønnegårdsvej 15, 1870 Frederiksberg C, Denmark; 2 Ø-Vet A/S, Køberupvej 33, 4700 Næstved, Denmark

**Keywords:** Post-weaning diarrhoea, Rotavirus, Enterotoxigenic *Escherichia coli*, pH, Rectal temperature, Pig

## Abstract

**Background:**

Recently, in-feed medicinal zinc has been phased out in pig production in the European Union. This makes updated knowledge about porcine post-weaning diarrhea (PWD) crucial. The objectives of the present study were to investigate (i) the clinical presentation of PWD in pigs housed in Danish herds that did not use medicinal zinc, specifically the prevalence of diarrhea and whether PWD was associated to clinical signs of dehydration or altered body temperature; (ii) which microorganism are associated to PWD; and iii) whether measurements of the fecal pH have a potential to be used diagnostically to differentiate between infectious etiologies in cases of PWD.

**Results:**

The prevalence of diarrhea varied considerably between the outbreaks in the nine studied herds (median = 0.58, range = 0.10; 0.94). In a cross-sectional design (n = 923), diarrhea was associated with reduced rectal temperature and alkaline feces. Diarrhea was also associated with observably reduced skin elasticity, possibly indicating dehydration. In both diarrheic case pigs (n = 87) and control pigs (n = 86), the presence of *Brachyspira pilosicoli*, *Clostridium perfringens*, *Cryptosporidium* spp., *Cystoisopora suis,* enterotoxigenic *Escherichia coli*, *Lawsonia intracellularis*, porcine circovirus types 2 and 3, rotavirus A, B, C, and H, *Samonella enterica spp. enterica*, and *Trichuris suis* was described. PWD was associated with high levels of enterotoxigenic *E. coli* shedding (odds ratio versus no *E. coli* detection = 4.79 [CI 1.14; 12.62]). Diarrhea was associated with high levels of rotavirus A shedding (odds ratio versus no/low rotavirus A = 3.80 [CI 1.33; 7.97]). The association between microbiological findings in diarrheic pigs and fecal pH was negligible.

**Conclusions:**

Enterotoxigenic *E. coli* was confirmed to be a cause of PWD; however, cases of PWD where enterotoxigenic *E. coli* was not detected in high levels occurred commonly, and this adds to the increasing evidence suggesting that PWD is not necessarily a result of enteric colibacillosis. Rotaviral enteritis might be a differential diagnosis of PWD. pH-measurements cannot be used to differentiate between differential diagnoses for PWD.

**Supplementary Information:**

The online version contains supplementary material available at 10.1186/s40813-023-00325-x.

## Introduction

Post-weaning diarrhea (PWD) is a common multifactorial clinical condition in intensive indoor pig production [1]. Adding high doses of medicinal zinc to the weaner diet has been used to prevent PWD [2–4]. Medicinal zinc co-selects for antimicrobial resistance, and most of the zinc is excreted with the feces. Through manuring, it accumulates in the soil, which is an environmental hazard [5]. As a result, the European Union prohibited the use of medicinal zinc as of June 2022 [6]. This has revitalized PWD as an important condition. Thus, it is crucial for veterinarians and pig producers to have up-to-date knowledge of the causes, clinical manifestations, and diagnostics of PWD outbreaks in herds weaning without medicinal zinc.

The multifactorial nature of PWD entails that factors related to the resilience of the pigs, management, housing, and feeding predispose for the clinical manifestation of infections with specific infectious agents [7–10]. Enterotoxigenic *Escherichia coli* (ETEC) is characterized by the ability to produce at least one of the enterotoxins: heat labile toxins (LT), heat stabile toxin type A (STa), or heat stabile toxin type B (STb), as well as having fimbriae, typically F4 or F18, allowing adherence to the intestinal epithelium [11–13]. ETEC is traditionally considered the main infectious etiology of PWD [13]. This perception is also prevalent in practice, where Danish veterinarians and stockpersons use *“post-weaning diarrhea”* and *“coli-diarrhea”* interchangeably [14]. However, a European study, analyzing diagnostic specimens from 873 pigs with PWD, did not detect ETEC in 40.4% of the herds (n = 113/280) [12]. PWD cases where ETEC was of limited importance have recently been documented in Danish pigs as well [1, 15]. Suggestively, these pigs could be affected by other enteric pathogens, such as rotavirus [1, 16, 17]. In older nursery pigs, non-infectious diarrheic conditions are common [18–20]. Similarly, causations related to post-weaning stress, feed composition, protein fermentation, and intestinal dysbiosis, i.e., with no infectious agents as contributing causes, have been suggested for PWD [21, 22]. This led us to hypothesize that other infectious etiologies than ETEC might be of importance and that cases with no infectious etiologies might be occur in outbreaks of PWD in Danish herds not using medicinal zinc in the feed.

To our knowledge, recent and systematic scientific descriptions of the clinical presentation of PWD under field conditions are limited. Outbreaks in Danish herds strongly associated with ETEC had been characterized in the 1970s, and the study described high mortality rates [23], which are not in accordance with the typical presentation reported from clinical practice today. In 1999, Canadian farmers reported dehydration, among other things, as a common feature of the PWD with the involvement of a certain ETEC strain [24]. Veterinary textbooks also include descriptions of the clinical presentation, and one book stated that fever is an occasional feature of post-weaning enteric colibacillosis [25].

When facing a case of PWD, information about the etiology qualifies veterinarians’ clinical decision-making, for instance regarding the relevance of antimicrobial therapy and the selection of an antimicrobial substance [26–28]. This is complicated when acknowledging that PWD have other etiologies than ETEC. Assigning the diagnosis enteric colibacilosis should rely on a quantitative interpretation of the test results [11]. Currently, real-time PCR quantification of the number of *E. coli* fimbria in a fecal sample is often used for this purpose [29], and therefore, it is relevant to know how to interpret quantifications of fimbria.

A major drawback of PCR and culture-based diagnostics is that the test results are not readily available, and rapid and cheap pen-side tests are in demand [30]. A candidate for a pen-side test could be measurements of the fecal pH. Researchers have recommended this measurement to differentiate between etiologies in cases of porcine [31] and human diarrhea [32] because the different pathophysiological processes are expected to cause certain alterations of the fecal pH. However, we found no empirical evidence supporting the practice.

In summary, the objectives of the present study were: (1) to describe the clinical presentation of PWD in pigs housed in Danish herds that did not use medicinal zinc, specifically the prevalence of diarrhea, and whether PWD was associated with clinical signs of dehydration or altered body temperature; (2) to investigate which microorganisms are associated with PWD in this population; (3) to establish how to interpret real-time PCR quantifications of *E. coli* fimbria in a fecal sample; and (4) to investigate the potential of fecal pH measurements for diagnostic differentiation between bacterial, viral, and non-infectious etiologies in cases of PWD.

## Material and methods

### Study design

**Fig. 1:**
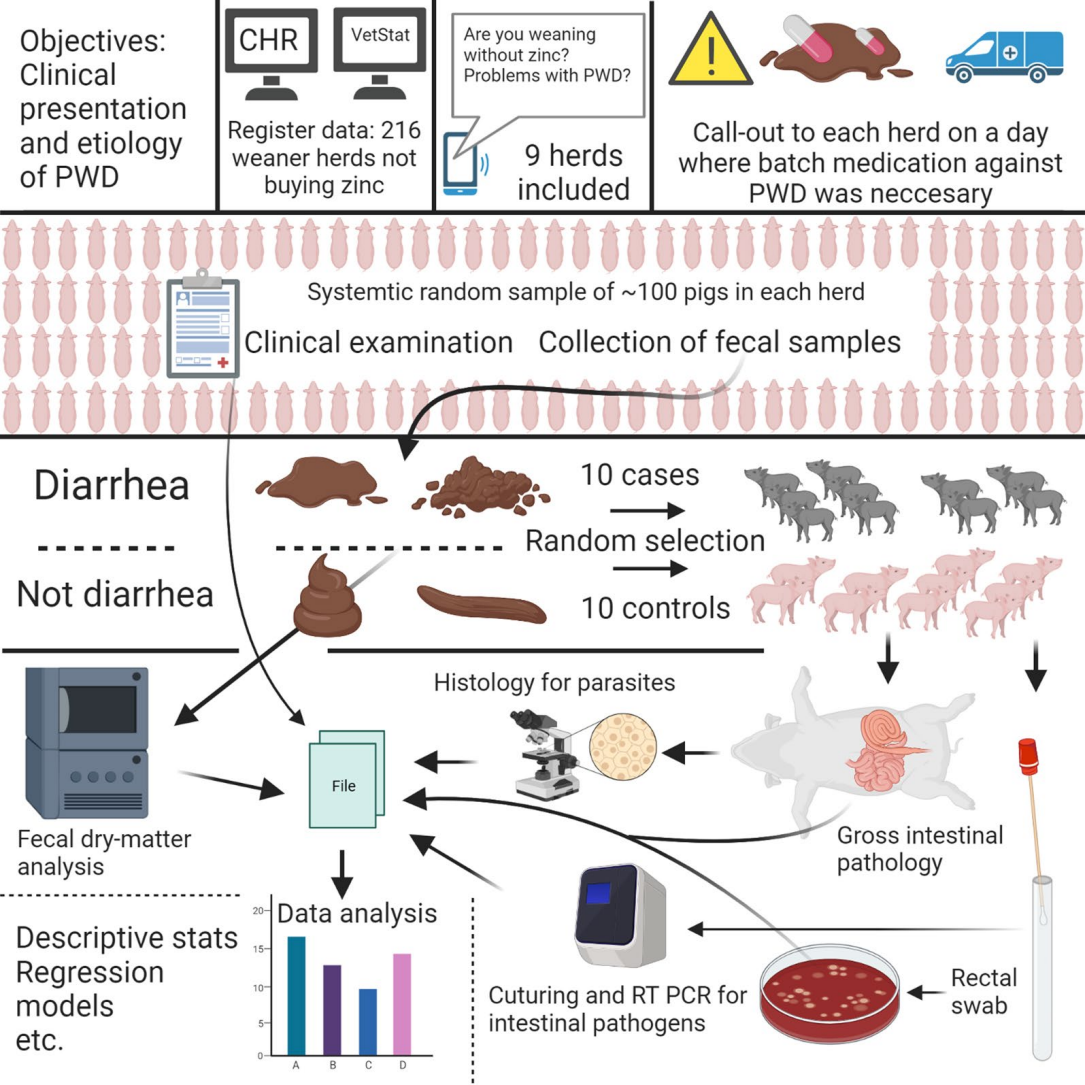
Graphical abstract of the methodology applied in an observational field study of post-weaning diarrhea. The illustration was created with BioRender.com. PWD: Post-weaning diarrhea. CHR: The Danish Central Husbandry register

The target population was Danish pigs weaned without in-feed medicinal zinc in intensive indoor productions. Objectives 1 and 4, i.e., clinical presentation of PWD and pH measurements, were studied in a cross-sectional investigation. Objectives 2 and 3, related to the microorganisms associated with PWD, were studied in a case–control investigation. The study design is summarized in Fig. 1 and described in detail below.

### Obtainment of study population

The study unit was individual pigs. The condition of interest was porcine PWD, defined as having loose or watery fecal consistency [33] during the first 14 days after weaning [1]. The pigs had to meet the following inclusion criteria; though, criteria E and F only applied to the case–control part of the study:

A. Was 0–14 days after insertion to the nursery unit.
B. Lived in a herd that did not use in-feed medical zinc.
C. Lived in a herd where initiation of antimicrobial flock medication was grounded in the clinical situation (i.e. not routinely/on a fixed time point).
D. Was part of a batch that had been assigned to antimicrobial flock medication due to PWD on the day of inclusion.
E. Had not received any antimicrobial treatment after weaning.
F. Delivered an adequate fecal sample during a clinical examination.

The pigs were included through a multi-stage sampling procedure with three stages described below. Additional details on sampling stages 1 and 2 can be found in Additional file 1.

#### Sampling stage 1: identification of herds

Herds meeting the following inclusion criteria were identified using the Danish Central Husbandry Register (CHR) [34], data on medicinal zinc purchases from the VetStat database [35], and telephone interviews:

1. Accommodated batches of at least 100 newly weaned pigs.
2. Did not use in-feed medical zinc.
3. Would possibly initiate an antimicrobial flock medication against post-weaning diarrhea in the study period.
4. Initiation of antimicrobial flock medication was based on the clinical situation (i.e., not routinely/at a fixed time point).
5. Was a production herd (i.e., not a breeding herd).
6. Were located within 2.5 h’ driving distance from the University of Copenhagen, Frederiksberg (See Additional file 1: Figure C).

The sixth criterion was set up to make acute callouts from Copenhagen to the PWD outbreaks feasible.

Herds enrolled in the study received detailed information and a request for the owner’s consent to the collection of data and usage for research purposes by e-mail. The herds were offered economical compensation for the euthanized pigs.

#### Sampling stage 2: selection of pigs for clinical examination

Nine herds recruited in sampling stage 1 were visited once on a day when the herd manager reported an outbreak of diarrhea deemed to require antimicrobial batch medication in more than 100 pigs. At each herd visit, a systematic random sample of 90 to 113 piglets was selected (see Additional file 2 defining the procedure) for a cross-sectional investigation, including clinical examinations and collection of fecal samples. The recorded variables are specified below (see *Clinical recordings* and *Fecal measurements* below).

#### Sampling stage 3: selection of cases and controls for further sampling and necropsy

Up to 10 apparent cases and up to 10 apparent controls were included, subjected to sampling for microbiology, and euthanized for necropsy at each of the examined outbreaks. Cases and controls were selected among all the examined pigs within a given herd meeting inclusion criteria E and F. The fecal consistency measured by the fecal dry matter content defined whether a pig was eligible as either a case or a control. However, the results of the fecal dry matter analysis (see below) could not possibly be available at the time of inclusion. Therefore, the pigs were initially categorized based on a fecal consistency score on a four-point scale [33] where 1 (firm) and 2 (soft and shaped) are not diarrhea, while 3 (loose) and 4 (watery) describe varying degrees of diarrhea. At each herd visit, 10 pigs were randomly [36] selected among all pigs that had delivered a fecal sample assessed to be diarrheic. Likewise, up to 10 pigs were selected among all pigs that had delivered a fecal sample assessed to be non-diarrheic. When the dry matter results were available, the status (case or control) was either confirmed or reallocated to the correct group. On two occasions, less than 10 apparent controls (Herd C) or cases (Herd G) were available to select from, and here all pigs with the given fecal score were included.

#### Sample size

Due to financial limitations, we aimed to include up to ten herds in sampling stage 1 and up to 20 pigs per herd in sampling stage 3. In sampling stage 2, the sample size was set to secure at least 10 cases eligible for sampling stage 3. We assumed that the diarrhea prevalence would be at least 15% at the time of batch medication (as previously suggested by [19]), and the lower bound of a Wilson 90% confidence interval for n = 100 and prevalence = 15% is 10%. That is, 10 cases would be available with 90% certainty in outbreaks with a low prevalence of diarrhea. We did not consider that the diarrhea prevalence could be above 85%, thus leaving us with too few controls (as in herd C).

### Clinical recordings

The clinical recordings are specified in Table 1. All the clinical evaluations were observed by the first author (EØE), except diarrheic soiling of the hind part, which was observed by four different technicians supervised by the first author (EØE). The rectal temperature was measured with a Digi-temp large animal thermometer (Jørgen Kruuse A/S, Langeskov, Denmark). Skin elasticity was measured by pinching and releasing the hairless area of skin caudal to the right pinnae. The clinical recordings were registered on paper sheets and typed into Microsoft Excel 2016 [37] twice. Differences between the two datasets were corrected, and a final check of a random sample revealed no typing errors.

**Table 1:** Descriptions of variables collected during a clinical examination of the pigs

Variable	Scale of measurement	Observations
Treated with antimicrobials after weaning	Dichotomous	No (0), Yes (1)
Diarrheic soiling of the hind part	Dichotomous	No (0), Yes (1)
Rectal Temperature	Discontinuous	32.0 to 42.0 °C (0.1 °C accuracy)
Skin elasticity	Ordinal	Normal (0), mild-moderately reduced (1), Markedly reduced (2)
Sunken eyes	Dichotomous	No (0), Yes (1)

### Fecal measurements

The variables collected for the fecal samples are listed in Table 2. The fecal samples from all pigs during sampling stage 2 were obtained from the rectum using digital manipulation, clinically evaluated, and stored in a plastic container [38]. The pH was measured at the herd visit after all clinical examinations had been performed. This practice sought to secure consistency in the scoring, and to minimize any biasing influence from the clinical impression of the pig. The scoring systems (consistency, deposits, and color) are displayed in Table 2. The fecal consistency was assessed as previously defined [33, 39].

**Table 2:** Variables measured to describe the fecal samples

Variable	Scale of measurement	Levels
Fecal sample	Nominal	Delivered a sample, sparse sample, no sample
Clinical assessment of the fecal consistency	Ordinal	1—Firm, 2—Soft and shaped, 3—Loose, 4—Watery
Fecal dry matter	Percentage	0 to 100%
Fecal color score	Nominal	Brown, black, grey, white, green, yellow
Fecal deposit score	Nominal	Blood, mucus, fibrin, necrotic debris
Fecal pH	Discontinuous	0; 14

As suggested in a standardized protocol for similar measurements in horse feces [40], the pH was measured directly in the fecal samples. We used a Seven2Go™ pH-meter (Mettler-Toledo A/S, Glostrup, Denmark) with three-step calibration with technical buffer at pH 4.01, pH 7.00, and pH 10.01 (± 0.03 pH) (Producer: WTW) and an InLab®Solids Go-ISM PRO IP67 electrode (Mettler-Toledo A/S), except in Herd A where an InLab® Expert Go-ISM electrode (Mettler-Toledo A/S) was used.

### Fecal dry matter analyses

Upon arrival at the University of Copenhagen, Frederiksberg, the fecal samples were stored at 4 °C overnight. The following day, the dry matter percentage was estimated by drying the fecal samples at 70 °C to 75 °C until constant weight (> 12 h). Thresholds for the corresponding fecal consistencies [33] were established as previously described [39] and are displayed in Table 3. The analysis indicated the dry matter estimates from Herd F were erroneous/biased, and therefore they were omitted.

**Table 3:** Thresholds for the fecal dry matter content (%) defining the qualitative description of fecal consistency

Fecal consistency (score)	Fecal dry matter content (%)
Firm and shaped (1)	> 22.7
Soft and shaped (2)	> 15 ≤ 22.7
Loose (diarrhea) (3)	> 9 ≤ 15
Watery (diarrhea) (4)	0 ≤ 9

### Microbiology

An additional fecal sample was collected from the rectum of all case and control pigs using a sterile cotton swab (OneMed, Helsinki, Finland) that was immediately placed in tubes with 5 of mL sterile phosphate buffered saline (PBS) solution [41]. The samples were transported to the laboratory in cooling boxes with ice and analyzed to detect intestinal pathogens that we hypothesized could be involved in the causation of PWD. The microbiological laboratory diagnostics are described in detail elsewhere [41]. In brief, we used culture-based methods supplemented by matrix-assisted laser desorption-ionization time-of-flight (MALDI-TOF) and PCR genotyping to detect *Clostridium perfringens, E. coli (ETEC and non-ETEC), and Samonella enterica spp. enterica* (*S. enterica*) serotypes*.* A high-throughput real-time PCR platform, BioMark HD (Fluidigm, San Francisco, California), and a 192.24 dynamic array integrated fluidic circuit system (Fluidigm) were used to quantify *Brachyspira pilosicoli*, *E. coli* fimbrial types F4 and F18, *Lawsonia intracellularis,* porcine circovirus type 2, porcine circovirus type 3, rotavirus A, rotavirus B, rotavirus C, and rotavirus H [42]. For a complete description of the procedure, we refer to the paper relating the microbiology to the pathological lesions [41]. Rotavirus B, C, and H were not part of the previously described platform [42], and the primer and probe sequences will be published elsewhere.

Herd C used a live oral vaccine (Coliprotec F4/F18, Elanco), and it is possible to culture the vaccine strains from vaccinated animals. Therefore, the O and H serotypes were obtained from the producer and also predicted from whole genome sequencing for six of the *E. coli* isolates from Herd C to verify that we had not cultured the vaccine strains. DNA isolation, sequencing, and sequence analysis for this part were done as described elsewhere [43].

The extent of the growth of hemolytic *E. coli-like* colonies was estimated semi-quantitatively [1, 41]. This information was missing for pigs from Herds E and F. This precluded the essential quantitative interpretation of ETEC diagnostics based on the culture results [11]. In these instances, we made imputations of the abundance of hemolytic *E. coli* (low or high) by using the established cut-off (see Results) of 17,500 F18 copies detected by real-time PCR.

### Pathology

The selected cases and controls (sampling stage 3) were stunned with a captive bolt and euthanized by exsanguination as the last procedure during a herd visit. The pigs were transported to the University of Copenhagen, Frederiksberg, and stored at 4 °C until the next morning, where a post-mortem exam focusing on the gastrointestinal tract was performed [41]. The intestines were removed from the abdominal cavity, and the appearance and content of all segments of the intestine were inspected. Tissue samples for histological evaluation were harvested from the mid-jejunum, mid-ileum, the apex of the colon spiral, lymphonodi jejunales, and lymphonodi colici. The procedures are described in detail elsewhere [41]. For the present objectives, the gross and histological examinations were aimed at detecting the presence of *Cryptosporidium* spp., *Cystoisopora suis*, and *Trichuris suis*. All post-mortem procedures were blinded, *i.e.*, the pathologists were not provided any information regarding the clinical recordings or fecal measurements.

### Specific research questions and statistical methods

Common descriptive statistics were used to summarize the data in multiple ways. Below, we describe the more advanced statistical work under sub-headings specifying the research question it addressed. All statistical work was performed in Stata IC 16 [44], except for one instance that is further specified below. All likelihood estimations of models were performed using maximum likelihood estimation, and model diagnostics were performed as described in [45]. From logistic models, we report the odds ratios and 95% confidence intervals. Marginal predicted probabilities with 95% confidence intervals were made from logistic models using linear predictions transformed with an inverse logit function using the command written in [46]. All reported confidence intervals are 95%, and we commonly abbreviate this “CI”.

#### What is the prevalence of post-weaning diarrhea on the day of antimicrobial batch-medication initiation in Danish herds weaning pigs without medicinal zinc?

The true prevalence of diarrhea within each outbreak was estimated. Some pigs delivered a fecal sample sufficient to accurately estimate the dry matter content, while others delivered a sparse or no fecal sample. This gave us the problem of missing values not-at-random when assigning a diarrhea status to the pigs. However, all pigs had recordings of diarrheic soiling of the hind part. Therefore, we applied a Bayesian method, taking assumed diagnostic test accuracies into account when estimating the true prevalence [47]. We wrote the code (see appendix in [47]) as a program in Stata IC 16 and validated it against the data and results described in the original paper and an online estimator where the method is available [48]. We assumed no errors in diarrhea diagnoses assigned by fecal dry matter content estimation. Evidence indicates some uncertainty when assigning a diarrhea diagnosis based on a clinician’s evaluation of a fecal sample [49]; nevertheless, rather few pigs were given the diagnosis this way, so for simplicity, we also assumed no errors for this method. Based on data from the present study, we assumed a sensitivity of 74.6% (95% CI 70.2; 78.7) (beta distribution: α = 302, β = 103) and a specificity of 83.3% (95% CI 78.8; 87.2) (beta distribution: α = 248, β = 50) when using the presence of diarrheic soiling of the hind-part as a diagnostic criterion. A prior prevalence distribution was formulated as a beta distribution (α, β) with α equal to 1 + the number of pigs with diarrhea based on the dry matter content or clinical assessment of the sparse fecal sample, and β equal to 1 + the number of pigs without diarrhea based on the dry matter content or clinical assessment of the sparse fecal sample. This prior prevalence distribution was then updated with pigs that lacked a fecal sample but had recordings of diarrheic soiling of the hind part, and thereby a posterior distribution was simulated [47]. We report medians with 95% credible intervals from the posterior distributions and present them in a bar chart.

#### What is the association between enteric infections and pigs displaying post-weaning diarrhea?

Some microorganisms were considered unlikely to cause PWD. Firstly, we had low prior expectations of their virulence, and additionally they were rarely detected (< 5% [n < 5] of the cases). For the more frequently occurring microorganisms, a set of simple logistic regressions was fitted to explore the apparent association between the microorganism (explanatory variable) and PWD (outcome variable). Based on these analyses and prior knowledge, rotavirus A, ETEC and certain *S. enterica* serotypes appeared to influence the probability of PWD. To disentangle the common appearance of mixed infections and account for the herd effect, the relevant pathogens were included as fixed effects in a multilevel logistic model with fecal consistency as a dichotomous variable as the outcome (diarrhea/not diarrhea). Our target estimate was the association, possibly representing the direct effect of each of the three pathogens. When drawing directed acyclic graphs to aid the model building [50], more than one possible causal relation between the three pathogens and PWD seemed probable to us. Thus, we were not confident in a certain causal diagram. However, irrespective of which of our directed acyclic graphs described the true state of nature, they coherently indicated that when estimating the association possibly representing the direct effect of a given pathogen, it was appropriate to adjust for both of the two other pathogens. The fixed effects were organized as shown in Table 4. The functional form of the relationship between the rotavirus A shedding cycle threshold (Ct) values from the real-time PCR and the odds of having diarrhea was first assessed by plotting the log-odds of diarrhea against the inverse Ct-values and adding a locally weighted scatterplot smoothing [51]. When modeling rotavirus A as a four-level categorical variable, using the quartiles of the Ct-value as cut-offs, the odds were similar for the three lowest quartiles (OR ≈ 1). Hence, we merged the three into one (Table 4), i.e., we dichotomized the variable leaving the upper quartile as “High rotavirus A (Ct < 9.23)”. *E. coli* had five possible levels based on the definitions described in [1]. As likelihood-based models converged poorly, Bayesian estimation was performed. We used 10,000 burn-in samples, and the sample size was set to 1,400,000. The latter was determined by assessing the effective sample sizes, and by computing Raftery-Lewis diagnostics [52] and Brooks-Draper diagnostics in MLwin version 2.36 [53, 54] aiming to get the mean of the posterior distributions correct with two digits with 95% certainty. We report the median odds ratios with 95% credible intervals from the model.

**Table 4:** Organization of fixed effects describing the presence of microorganism in fecal samples

Variable	Levels
*E. coli*^*a*^	0: No *E. coli* growth 1: Low non-ETEC hemolytic *E. coli* 2: High non-ETEC hemolytic *E. coli* 3: Low ETEC 4: High ETEC
Rotavirus A (RVA)	0: No or low RVA detected (Ct > 9.23) 1: High RVA (Ct < 9.23)
*Salmonella enterica*	0: Not detected 1: Detected

^a^See [1] for definitions of the *E. coli* categories

The fixed effects were included in a multilevel logistic model exploring the association between the assumed infectious etiologies and post-weaning diarrhea (PWD), and two multilevel linear models exploring the association between the abundance of assumed infectious etiologies and the rectal temperature and the pH of the feces collected from pigs with PWD.

#### What is the best available cutoff, if using real-time PCR quantification of fimbria antigens to predict at least 50% growth of fimbria positive hemolytic *E. coli* on blood agar*?*

We defined the best available cutoff as the point where the maximal sensitivity + specificity was reached. A non-parametric receiver-operating characteristic (ROC) curve [55] was estimated. The number of F18 DNA-copies was the classification variable, and a dichotomous recording of the culture results was the reference variable, i.e., the presence of ≥ 50% growth of hemolytic *E. coli* with F18. The sensitivity and specificity with associated 95% confidence intervals were estimated for the selected cut-off using the command written in [56]. Abundant growth of hemolytic *E. coli* with F4 was only observed three times, and thus no ROC curve could be fitted.

#### Is detection of fimbria predicting that *E. coli* isolates are enterotoxigenic?

We estimated the probability with 95% confidence intervals [57] of *E. coli isolates* being enterotoxigenic if either F4 or F18 had been detected*.* For a quantitative comparison to the literature, we searched the Web of Science Database for studies with similar data obtained from pigs housed in intensive indoor productions in Europe. We were familiar with a pre-print containing such data as well [15]. We harvested data from the studies and estimated probabilities as described above.

#### Is post-weaning diarrhea and altered rectal temperature associated?

We fitted multilevel logistic regression with the fecal consistency as a dichotomous variable as the outcome (not diarrhea/diarrhea) and the rectal temperature (below normal [≤ 38.8 °C], normal [38.9 to 39.8 °C], above normal [≥ 39.9 °C]) as a fixed effect. The cutoffs for the three-level categorization of rectal temperature were decided based on prior knowledge of the normal body temperature of weaned pigs collected from two textbooks [58, 59] together with a locally weighted scatterplot smoothing [51] of the log-odds of diarrhea plotted against the rectal temperature.

#### Are certain infectious etiologies associated with the rectal temperature in pigs suffering from post-weaning diarrhea?

We fitted a mixed linear model with rectal temperature as the outcome, binary registrations of assumed etiologies (rotavirus A, ETEC, or *Salmonella*) as fixed effects (Table 4), and herd as a random effect. The analysis was restricted to pigs suffering from diarrhea. We assumed a possible mediator: that the etiology had an effect on the consistency of the feces and that the consistency had an effect on the temperature. Hence, we adjusted for fecal consistency (watery versus loose) in the model to estimate the association, possibly representing the direct effect [50]. Linear predictions with confidence intervals were reported from the model.

#### Is reduced skin elasticity associated with diarrhea?

As markedly reduced skin elasticity was rarely observed (0.87%, n = 8), we used a dichotomized version of skin elasticity (not reduced/reduced) as the outcome in a multilevel logistic model. The fecal consistency (1–4) was a fixed effect, and herd was added as a random effect.

#### Is the assumed etiology associated to the fecal pH in pigs suffering from post-weaning diarrhea?

The fecal pH was plotted against the fecal dry matter percentage. The trend in this scatterplot was visualized using a local polynomial smoothing (third-degree Epanechnikov function) with a 95% confidence interval. Measurements of fecal samples weighing less than 1 g and originating from Herd F were excluded from this plot. We fitted a mixed linear model with pH as the outcome, assumed etiologies as fixed effects (Table 4), and herd as a random effect. A binary variable describing the fecal consistency (loose or watery) was added to the model as this was assumed to be a mediator, and we wanted to estimate the association, possibly representing the direct effect [50] of the assumed etiology on the fecal pH. The model estimation was restricted to pigs suffering from diarrhea.

## Results

### Herd descriptions

Some central characteristics of the nine herds are described in Additional file 3: Table A. The median batch size was 432 (range 139 to 1135). Five of the herds were weaning to 30 kg production, and the examined PWD outbreaks most commonly (n = 6/9) occurred four days after the batch had been inserted into the nursery unit. The herds raised pigs with DanBred genetics, except Herd K which used Danish Genetics. Herd C did not know the genetics of the animals. The pigs in Herd E and Herd F originated from the same sow unit (owned by the producer of Herd E), and Herd F bought the heaviest pigs in each batch.

### Data completeness

In total, 924 pigs were clinically examined, of which 699 (75.6%) delivered a fecal sample, including 47 (5.1%) samples that were sparse (< 1 g). It was possible to measure the pH in 619 of the fecal samples. Inclusion criterion E was not met by 15 pigs; 10 pigs had received individual antimicrobial treatments (n = 1 lacked a fecal sample as well); in herd K, some of the medicated water for the batch medication that was meant to be initiated immediately after our data collection had been spilled in a pen with five included pigs (n = 1 lacked a fecal sample as well), and we could not preclude that the pigs had ingested it. All cases and control pigs (n = 173) were assigned a fecal consistency based on a dry matter estimate, except 31 pigs (including the 20 pigs from herd F), where the clinician’s assessment of the fecal sample was used. One recording was missing of the rectal temperature, the skin elasticity, antibiotic use, sunken eyes, and sex. In Herd A, seven pigs lacked genotyping of highly abundant *E. coli* isolates, and 40 pigs (all the pigs in Herds E and F) lacked semi-quantitative estimates of the number of *E. coli*. Imputations were made as described in *Materials and methods.* In Herd A, growth descriptions were similar (100% hemolytic *E. coli-*like colonies) for all pigs (n = 20). However, for two of these, MALDI-TOF did not confirm that they were E. coli (no species identified), and we omitted them from the analysis (i.e., ETEC diagnostics changed to missing values).

### Clinical presentation of the post-weaning diarrhea outbreaks

**Fig. 2:**
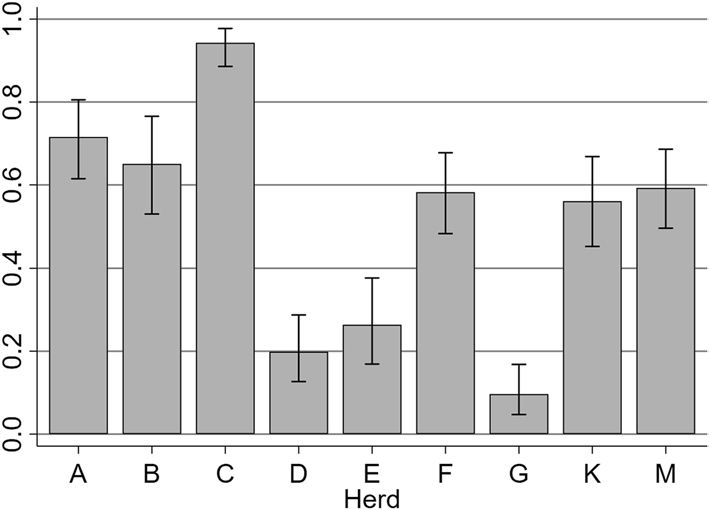
The true prevalence proportion (with 95% credible interval) of post-weaning diarrhea. Data was collected at the time of initiation of antimicrobial batch medication in nine Danish herds weaning without medicinal zinc

**Fig. 3:**
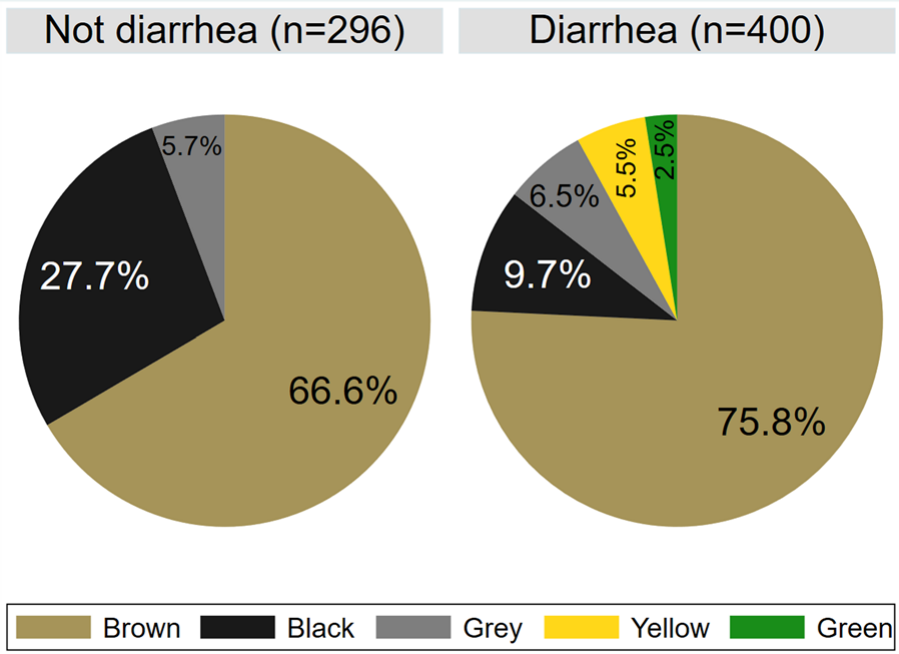
The color of the non-diarrheic feces (left) and diarrheic feces (n = 400) (right). The data was collected from pigs in outbreaks of post-weaning diarrhea in nine Danish commercial indoor herds weaning without medicinal zinc

Across the nine herds, 184 (19.9%) pigs had firm feces, 112 (12.1%) had soft and shaped feces, 131 (14.2%) had loose feces, 272 (29.4%) had watery feces, and 225 (24.4%) of the pigs were not assigned a fecal consistency. As seen in Fig. 2, there was a considerable variation in the estimated true prevalence of diarrhea in the nine outbreaks. The color of the fecal samples is displayed in Fig. 3. Brown was common across all fecal consistencies, while yellow and green were only observed in diarrheic samples. In the diarrheic samples, we observed absence of deposits in 92.5% (n = 370), mucus in 4% (n = 16), fibrin in 2% (n = 8), both mucus and fibrin in 0.75% (n = 3), blood in 0.5% (n = 2), and both blood and mucus in 0.3% (n = 1), Necrotic debris was not observed, and no deposits were observed in non-diarrheic samples.

**Fig. 4:**
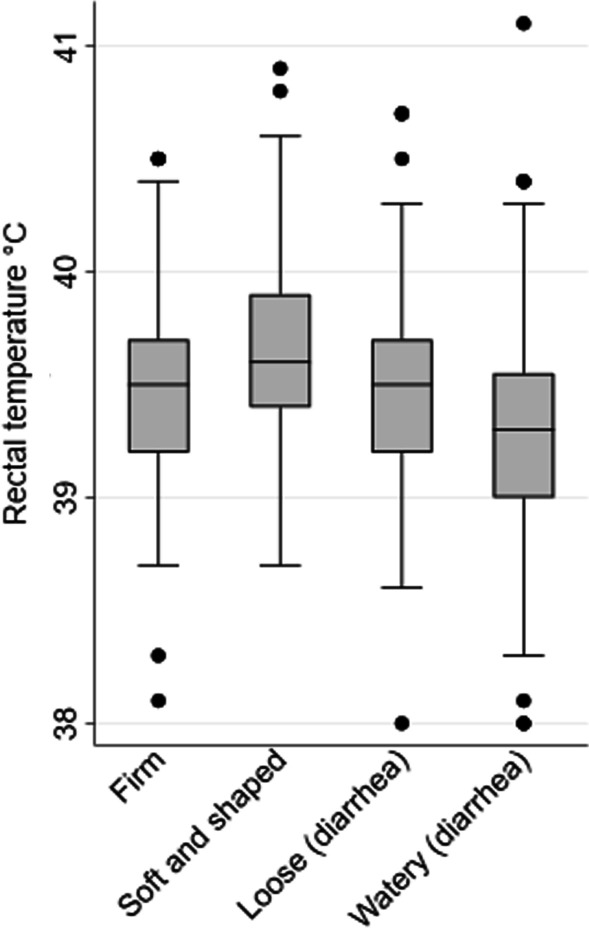
The rectal temperature (°C) of newly weaned pigs (n = 698) sorted by fecal consistencies. The data displayed in the boxplots was collected in outbreaks of post-weaning diarrhea in nine Danish commercial indoor herds weaning without medicinal zinc

**Fig. 5:**
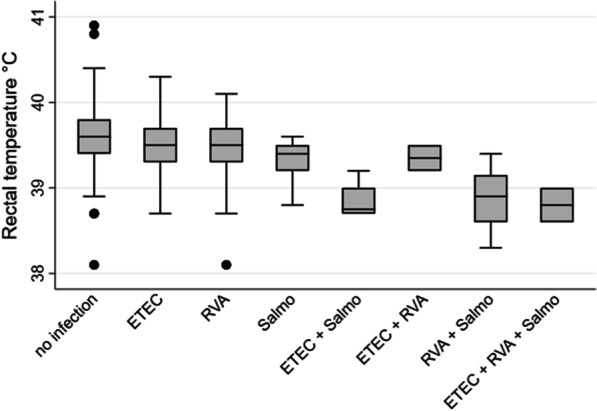
Rectal temperature (°C) of pigs (n = 87) suffering from post-weaning diarrhea sorted by assumed etiology. The data displayed in the boxplots was collected in outbreaks of post-weaning diarrhea in nine Danish commercial indoor herds weaning without medicinal zinc. no infection: Pathogens were not detected or only detected at low level. ETEC: High levels of Enterotoxigenic* E. coli*. RVA: High Levels of rotavirus A. Salmo:* Salmonella enterica*

Reduced rectal temperature (≤ 38.8 °C) was recorded in 91 pigs (9.9%), and increased rectal temperature (≥ 39.9 °C) was recorded in 132 (14.3%) of the pigs. The rectal temperatures are displayed in boxplots in Fig. 4 sorted by fecal consistencies (1–4) and in Fig. 5 sorted by assumed etiology in pigs suffering from diarrhea. As seen from the estimates in Table 5, reduced body temperature was associated with diarrhea. It is evident from Fig. 4 that the association was strongest in pigs with watery diarrhea and less distinct in pigs with loose fecal consistency. Not having diarrhea was associated to increased body temperature (Table 5), and this association was driven by pigs with soft and shaped feces (Fig. 4). The predicted probability of diarrhea was 82.7% (CI 57.7; 94.3) for pigs with reduced rectal temperature, 57.8% (CI 36.4; 76.6) for pigs with normal rectal temperature, and 45.1% (CI 23.9; 68.2) for pigs with increased rectal temperature. The predicted mean temperature was 38.85 °C (CI 38.56; 39.14) for diarrhea cases with detection of *S. enterica,* and this was lower than for cases with no detection of this pathogen, for which the predicted mean was approximately 39.47 °C (see Table 5).

**Table 5:** Odds ratios of pigs (n = 698) having post-weaning diarrhea given different rectal temperatures

	Odds ratio	95% CI
Baseline odds (constant)	1.37	0.57; 3.28
Normal rectal temperature	Baseline	
Reduced rectal temperature	3.49	1.34; 9.07
Increased rectal temperature	0.6	0.37; 0.97
Variance, random effect: herd	1.68	0.62; 4.57

As displayed in Table 6, 127 pigs (32%) with diarrhea had reduced skin elasticity. The odds of reduced skin elasticity increased as the fecal consistency became more watery (Table 6). Sunken eyes were observed 18 times: in 14 pigs with diarrhea, three pigs without diarrhea, and in one pig with no fecal sample delivered (Table 7).

**Table 6:** Association between reduced skin elasticity and fecal consistency (n = 699)

Fecal consistency/Skin elasticity	Reduced skin elasticity	Odds ratio for reduced skin elasticity	95% CI	Predicted %	95% CI
Firm	11 (6%)	Baseline		4.0	1.4; 11.0
Soft and shaped	10 (9%)	1.96	0.75; 5.09	7.5	2.6; 19.7
Loose (diarrhea)	24 (18%)	3.01	1.27; 7.16	11.1	4.4; 25.3
Watery (diarrhea)	103 (38%)	9.33	4.23; 20.62	27.9	13.4; 49.1

The data was collected in outbreaks of post-weaning diarrhea in nine Danish commercial indoor herds weaning without medicinal zinc. Odds ratios and predicted percentages were estimate from a multilevel logistic model. The variance estimate for the random effect (herd) was 1.6 (95% CI 0.56; 4.75)

**Table 7:** The predicted rectal temperature (°C) of diarrheic pigs (n = 85) with different assumed etiologies

Assumed etiology	Mean	95% CI
No/low infection	39.46	39.35; 39.57
*High* enterotoxigenic* E. coli*	39.46	39.30; 39.61
High rotavirus A	39.48	39.34; 39.62
*Salmonella enterica*	38.85	38.56; 39.14

The data was collected in outbreaks of post-weaning diarrhea in nine Danish commercial indoor herds weaning without medicinal zinc. The herd level variance (random effect) was 0, and the random error term was 0.12 (0.09; 0.17). Fecal consistency (watery versus loose) was adjusted for in the model

ETEC: High levels of Enterotoxigenic *E. coli.* RVA: High Levels of rotavirus A. Salmo: *Salmonella enterica.*

### Microbiology and pathology

Table 8 displays the microbiological findings divided by herd and fecal consistency (diarrhea/not diarrhea). ETEC was detected in high levels in 23 out of 87 pigs with diarrhea. ETEC could not be detected in high levels in any diarrheic pigs in four of the herds. Rotavirus A and C were commonly detected. The serotypes of *S. enterica* were typically (n = 11/13) Typhimurium (Herd C and D), but the Derby serotype also occurred (2/13) (Herd K). *Brachyspira pilosicoli* and *Lawsonia intracellularis* were not detected by real-time PCR, and *Clostridium perfringens* type C was not detected by anaerobic culturing, while combined culture and PCR detected *C. perfringens* type A in 4 pigs with diarrhea. Macroscopic inspection and histopathological evaluation of the intestines did not reveal signs of *Cryptosporidium* spp.*, Cystoisospora suis.* or *Trichuris suis*. The histopathological evaluations revealed the protozoan *Balantidum coli* in the colon of 15 pigs.

**Table 8:** Pigs (n = 173) with and without post-weaning diarrhea according to pathogens detected in their fecal samples

Herd	A		B		C		D		E		F		G		K		M		Total	
No. of pigs without (-) and with diarrhea ( +)	−	+	−	+	−	+	−	+	−	+	−	+	−	+	−	+	−	+	−	+
	10	10	8	12	5	9	10	10	12	8	10	10	12	7	10	10	9	11	86	87
Rotavirus A (high)	0	0	2	8	2	4	3	6	3	5	1	0	1	4	1	3	1	0	14	30
Rotavirus A (low)	7	9	5	4	3	4	7	4	9	3	4	3	6	1	8	7	8	11	57	46
Rotavirus C	7	7	7	6	4	5	2	2	7	3	6	8	6	1	4	3	8	8	51	43
High ETEC	8^a^	10^b^	0	0	1	5^c^	1	2	1	0	0	0	0	0	0	4	0	2^c^	11	23
Low ETEC	0	0	0	1	0	0	3	1	0	1	4	3	0	1	4	4	1	1	12	12
High non-ETEC EC	0	0	0	0	0	2	0	0	3	2	2	2	0	0	0	0	3	3	8	9
Low non-ETEC EC	0	0	0	0	0	0	2	1	3	3	4	5	3	1	1	0	5	4	18	14
*Salmonella enterica*	0	0	0	0	3	8	2	1	0	0	0	0	0	0	1	1	0	0	6	10
*Balantidum coli*	0	0	1	1	0	0	2	0	2	1	2	1	1	1	0	1	0	2	8	7
Rotavirus B	1	0	1	2	1	2	0	0	1	0	0	0	0	0	0	0	0	0	4	4
Porcine circovirus 2	0	0	0	0	0	0	0	0	1	0	0	0	0	1	0	0	5	0	6	1
Porcine circovirus 3	2	1	1	2	0	0	0	0	0	0	0	0	0	0	0	0	0	0	3	3
*Clostridium perfringens* type A	0	0	0	0	0	0	0	2	0	0	0	0	0	0	0	0	0	0	0	2
Rotavirus H	0	0	0	0	0	0	0	0	0	0	0	0	0	0	1	0	0	0	1	0

The numbers of pigs are sorted by nine Danish herds (A-M) weaning without medicinal zinc

^a^Missing values for two pigs, and imputations made for three pigs

^b^Imputations made for four pigs

^c^Missing values for one pig

ETEC: Enterotoxigenic *Escherichia coli*

Non-ETEC EC: hemolytic *Escherichia coli* that are not enterotoxigenic

The genotypes of the *E. coli* isolates are displayed in Table 9. The most prevalent non-ETEC genotype was F18, and F18 + Lt + StB was the most prevalent ETEC type. The probability of hemolytic *E. coli* isolates being able to produce enterotoxins was 86% (CI 42; 100) (n = 6/7) and 56% (CI 45; 67) (n = 45/80) for isolates positive for F4 and F18, respectively. Data on whether *E. coli* isolates were able to produce enterotoxins when carrying either the F18 (n = 590/955) or the F4 (n = 589/627) genes was successfully collected from 12 European studies and analyzed (see Additional file 4: Table B and Figure D).

**Table 9:** Virulence factors genes in hemolytic *Escherichia coli* isolates from pigs (n = 164) with and without post-weaning diarrhea

Virulence factor genes	Diarrhea	Not diarrhea
F18	18	14
F18 + Lt + StB	10	10
F18 + STa	7	4
No fimbria, no toxin	4	8
F18 + ?	4	3
F4 + Lt + STb	4	1
F18 + Lt	4	1
F18 + STa + STb	3	4
F18 + STb	2	0
F18 + Vt1 + Vt2	1	2
F4 + STa + STb	1	0
F4	1	0
STb	0	1

The data was collected in outbreaks of post-weaning diarrhea in nine Danish commercial indoor herds weaning without medicinal zinc. Rows are sorted according to prevalence among diarrheic pigs, and subsequently, prevalence among non-diarrheic pigs

F4: Fimbria type 4

F18: Fimbria type 18

STa: Heat-stabile toxin type A

STb: Heat-stabile toxin type B

Lt: Heat-labile toxin

Vt1: Verotoxin type 1

Vt2: Verotoxin type 2

?: Missing values for the toxin genotypes

The best cut-off in real-time PCR for predictions of when ≥ 50% growth of hemolytic *E. coli* F18 by culture could be expected was between 15,036 and 19,902 copies, and we selected 17,500 copies as a compromise between the two. The area under the ROC-curve was 0.83 (CI 0.76; 0.91), the diagnostic sensitivity was 78.9% (CI 62.7; 90.4), and the specificity was 80.6% (CI 71.1; 88.1).

**Fig. 6:**
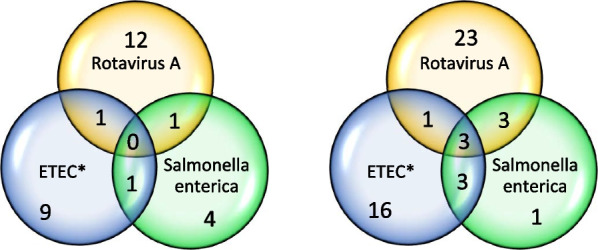
Number of pigs with single or mixed infections in pigs with and without post-weaning diarrhea. The left panel displays pigs without diarrhea (n = 86), and the right panel displays pigs with diarrhea (n = 87). Values for ETEC were missing for two pigs with diarrhea and two pigs without diarrhea

The mixed infections with ETEC, rotavirus A, and *S. enterica* in pigs with and without PWD are displayed in Fig. 6. In a substantial fraction (n = 36/87, 43%) of the pigs with diarrhea, none of these three pathogens were detected or were only detected at low levels. Table 10 displays the estimated odds ratios of diarrhea given the shedding of different types and levels of hemolytic *E. coli*, rotavirus A, and/or *S. enterica*. The estimates generally had a wide span of uncertainty, and diarrhea was associated with shedding high levels of ETEC (OR = 4.79 [CI 1.14; 12.62]) and rotavirus A (OR = 3.80 [CI 1.33; 7.97]). The low herd-level variance should be attributed to design factors; the outcome was almost balanced between herds due to the case–control design.

**Table 10:** Odds ratios of having post-weaning diarrhea given fecal shedding of certain microorganisms

	Odds ratio	95% credible interval	
Constant: baseline odds	0.41	0.20	0.79
No *E. coli*	Baseline		
Low hemolytic non-ETEC	1.61	0.44	3.82
High hemolytic non-ETEC	2.06	0.31	5.77
Low ETEC	1.84	0.42	4.53
High ETEC	4.79	1.14	12.62
Rotavirus A: no/low	Baseline		
Rotavirus A: high	3.80	1.33	7.97
*S. enterica*: no	Baseline		
*S. enterica*: yes	1.30	0.14	3.32
Variance: herd	0.05	0.001	0.47

The levels of enterotoxigenic *E. coli* (ETEC), non-ETEC hemolytic *E. coli,* rotavirus A and *Salmonella enterica spp.* detected in case and control pigs. Estimates are from a Bayesian mixed logistic model

### Fecal pH

**Fig. 7:**
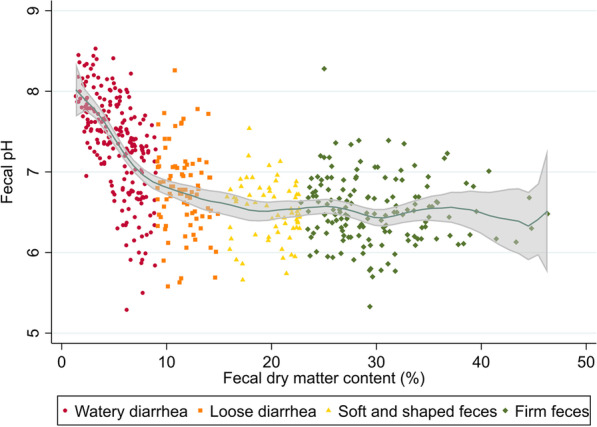
The fecal pH versus the dry-matter content in fecal samples from post-weaning pigs (n = 516). The polynomial line with associated confidence interval was smoothed using a third-degree Epanechnikov function. The cut-offs defining the color and shape of the markers in the plot are described in Table 3

As evident from Fig. 7, increased pH was observed in watery diarrhea, especially in the very watery samples (below approximately 5% dry matter), where the pH was markedly alkalized. The direct effects of the infectious etiologies on the fecal pH listed in Table 11 appeared to be absent or quite small.

**Table 11:** The associations between assumed etiologies and the fecal pH in pigs with post-weaning diarrhea (n = 82)

	Coefficient	95% CI	
Constant: baseline pH	6.79	6.45	7.13
Not high ETEC	Baseline		
High ETEC	− 0.21	− 0.53	0.11
Rotavirus: no/low	Baseline		
Rotavirus: high	− 0.06	− 0.32	0.21
*S. enterica*: no	Baseline		
*S. enterica*: yes	0.17	− 0.35	0.69
Variance: herd	0.17	0.06	0.52
Random error	0.21	0.15	0.29

The estimates are from a mixed linear model, and there is adjusted for fecal consistency (watery versus loose)

## Discussion

This study investigated the clinical presentation and the microorganisms that are associated with PWD in pig herds weaning without use of medicinal zinc in the feed.

The small sample size, especially in the case–control part of the investigation, makes strong conclusions impossible. We considered all herds that weaned without medicinal zinc in eastern Denmark in 2019, however, only a low number of herds fulfilled the inclusion criteria. As of June 2022, all European herds have had to change into weaning without medicinal zinc, and the nine herds included in this study might not be fully representative of the herds now going through this transition. Herds weaning without medicinal zinc and having problems with diarrhea, at the time we sampled herds for inclusion, possibly differ from the general population in some ways. For instance, herds with innovative owners may have been more likely to try out weaning without medicinal zinc, and herds experiencing severe problems (related to management and microorganisms) with diarrhea might have reintroduced the use of medicinal zinc.

### The clinical presentation of post-weaning diarrhea

The prevalence of diarrhea varied considerably in the nine PWD outbreaks studied in Danish indoor herds weaning without medicinal zinc in 2019. The prevalence of diarrhea was generally higher at the time of treatment initiation than previously described in outbreaks of diarrhea in older Danish nursery pigs [19].

PWD was not associated with fever, and pigs with PWD were at higher risk of having a reduced rectal temperature. Studies where the mean rectal temperature has been estimated in pigs experimentally challenged with ETEC have reported diverse findings. Two studies reported increased rectal temperatures the two first days after inoculation compared to unchallenged controls [60, 61]. In line with this, another study demonstrated a marked temperature increase (~ 40.5 C) 9 h after inoculation. This had, to a large extent, worn off (~ 39.6 C) already 24 h post-inoculation. Yet, other studies have reported reduced rectal temperatures in the challenged groups 1–3 days after inoculation [62] or overall for the whole study period of 10 days after inoculation [63]. The cross-sectional observations of the present study and the previously reported experimental trials could suggestively reflect that fever sometimes occurs in the early phase of PWD associated with ETEC. This acute phase is then followed by days with diarrhea and, depending on the severity of the diarrhea, this may be associated with reduced body temperature. In our analysis restricted to diarrheic pigs, *S. enterica* infection was associated with a lower rectal temperature, while rotavirus A and ETEC were not*.* This should be interpreted with great caution since *S. enterica* mainly occurred (n = 9/10) in mixed infections with ETEC and/or rotavirus A.

We found that the observed reduced skin elasticity was associated with diarrhea. A logical explanation is that diarrhea leads to loss of fluid and electrolytes and consequently dehydration with observable an reduction of the skin elasticity. Among the pigs with watery diarrhea, 38% were evaluated as having reduced skin elasticity. Thus, the proportion of pigs suffering from dehydration during PWD outbreaks might be substantial. This would underline the relevance of rehydration therapy during PWD outbreaks [64]. However, we were not able to blind the fecal consistency to the person evaluating the skin elasticity, since the clinical examination and fecal sample collection were performed concurrently. Thus, the association may be subject to observer bias elicited by confirmation bias. Future studies should rule out this possibility by collecting blinded observations or objective measures of dehydration (e.g., blood values).

### Infectious causes of post-weaning diarrhea

It is well established that ETEC causes PWD [11, 13]. In accordance with this, the present study found that PWD was associated with the shedding of ETEC at high levels (Table 11). A primary motivation for the present study was to determine how frequently cases of PWD with and without the involvement of ETEC occur. As we had hypothesized, cases and even complete outbreaks with no involvement of ETEC were observed, and in this small sample, quite commonly. This adds to the evidence indicating that diarrhea occurring in the immediate post-weaning period in intensive indoor pig production should not immediately be considered enteric colibacillosis [1, 12, 65].

Our data indicated that *E. coli* fimbriae type F4 often had the ability to produce enterotoxin. In contrast, *E. coli* fimbriae type F18 was frequently negative for enterotoxin genes. This is in accordance with previous European studies collecting similar data. When analyzing data from all studies collectively (Additional file 4: Table B and Figure D), the crude percentages of isolates able to produce enterotoxins were 94% (CI 92; 96) for F4 and 62% (CI 59; 65) for F18 [1, 12, 15, 43, 66–73]. This also corresponds well with the previous finding that the F4 clone circulating in Danish pig herds is clonal, toxigenic, and has remained stable over several decades, suggesting that F4 ETEC is a stable pathogen [43].

In a recent Danish cohort study, originating from the same project as the present study, the association between a high abundance of non-ETEC hemolytic *E. coli* and PWD occurrence was also estimated [1]*.* Both the cohort study and the present results indicated a weak association between PWD and the isolation of non-ETEC hemolytic *E. coli*. However, the span of uncertainty for the estimates was wide, and the true magnitude may likely be negative, null, or a stronger positive association. In an older experimental study, fimbria-positive and toxin-negative *E. coli* induced mild and transient diarrhea in some of the inoculated day-old piglets, while no diarrhea was seen in the negative control group [74]. In the same study, the group inoculated with toxin-positive *E. coli* experienced diarrhea at a higher rate and with a more severe presentation [74]. Conclusively, the virulence of non-ETEC hemolytic *E. coli* has not been thoroughly investigated; nevertheless, the available data vaguely indicates that the bacteria might have a minor ability to produce enteric disease with limited severity.

PWD was associated with shedding high levels of rotavirus A (Table 11). It is well-established that rotavirus A is an enteric pathogen that causes diarrhea in domestic animals, including pigs [75], but its significance as an agent causing PWD is less clear and may potentially be limited. Several publications describes presence of rotavirus in diagnostic specimens from diarrhea cases in pigs (e.g., [1, 76]), but evidence allowing conclusions about association or even causation (i.e. including non-diarrheic or unexposed controls) is sparse. Observational field studies have associated rotavirus A to PWD [16, 17] and demonstrated villus atrophy in situ [17]. In an experimental study, diarrhea was induced in weaned pigs by inoculating them with rotavirus A [77]. Contradicting this, naturally occurring rotavirus A infections did not induce diarrhea in the control group in an ETEC-challenge study [78] and in an experimental trial investigating the effect of creep feeding on PWD, rotavirus A occurred at the same frequency in diarrheic and non-diarrheic animals; yet, the virus only observed in low quantities [79] and the present study indicated that high levels of rotavirus A is required to induce diarrhea. Collectively, the literature and the present study suggests that rotavirus A may be an etiological agent of PWD, but the evidence of this is not strong. As rotavirus A is ubiquitous in the indoor pig production, and the prevalence peaks in the post-weaning period [80], is it important to further investigate its role as primary PWD pathogen by obtaining unbiased samples and analyzing specimens from control animals as well as diseased ones.

As previously discussed, our data included rather few pigs which were positive for *S. enterica,* and they primarily occurred in mixed infections originating from a single herd. Therefore, we cannot make sound conclusions on the clinical significance of *S. enterica* from the present study. A textbook describes *Salmonella enterica* serotype Typhimurium as an enteric pathogen causing diarrhea in pigs [81], and diarrhea has been induced in experimentally inoculated weaned pigs (e.g., see [82]). However, to our knowledge, the occurrence and clinical significance of *S. enterica* in field cases of post-weaning diarrhea have not been thoroughly investigated, and the present study highlights the need for this.

The number of pigs that were infected with *C. perfringens* type A*,* porcine circovirus 2, porcine circovirus 3, rotavirus B, and rotavirus H (Table 8) made it improbable to conclude about their role in PWD. However, their rare occurrence indicates that they are not important for PWD in Danish herds weaning without medicinal zinc. Rotavirus C occurred frequently (Table 8), but a crude association with PWD was absent. Literature reports the detection of rotavirus C in diagnostic samples from PWD (e.g., [76, 83]), but to our knowledge, no studies that allowed concluding on association or causation have been conducted. The present study appears to be the first to try to estimate the association to PWD for other rotavirus groups than A, and results indicate that rotavirus C, B, and H are not important etiologies of PWD. *Balantidum coli* was the only parasite detected, and this protozoan was not associated with PWD. The implications of this finding have been discussed elsewhere [41].

Almost all Danish herds are expected to be endemically infected with *L. intracellularis,* and *B. pilosicoli* is also commonly present [84, 85]. When endemic, shedding of these pathogens normally first occur later in the nursery period [86–88]. Accordingly, *L. intracellularis* and *B. pilosicoli* were not detected in any of our samples collected shortly after weaning.

A limitation of the present study is that we only investigated a selected set of microorganisms that we considered relevant based on prior knowledge. Denmark is declared free from porcine enteric coronaviruses [89], and we believe we have included the relevant microorganism in our protocol. Yet, it precluded the identification of novel infectious etiologies of PWD, including microorganism which have been hypothesized to be enteric pathogens in young pigs. For instance, Park and colleagues hypothesized that porcine kobuvirus may be an underestimated pathogen in this respect [90]. However, a recent review paper concluded that *“the sparse available evidence suggests that porcine kobuvirus is of limited clinical importance*” [91]. For porcine sapovirus, a fairly recent review paper concluded: *“Although the first porcine sapovirus was detected four decades ago, their role in causing pig diarrhea in the field remains undetermined*” [92].

### Interpretation of real-time PCR quantification of *E. coli* fimbria

Enteric colibacilosis may be diagnosed when liquid feces is observed together with a high abundance of ETEC in the intestine [11]. The latter may be supposed when culturing feces on blood agar exhibits 100% [11] or ≥ 50% [1, 41, 79] growth of hemolytic *E. coli* colonies that are confirmed to be ETEC by genotyping. However, real-time PCR could also be useful in this regard, and for F18, we evaluated how well it corresponded with the culture-based method; we established a cut-off and diagnostic sensitivity and specificity for predictions ≥ 50% growth of hemolytic *E. coli* F18 on blood agar. The ROC curve indicated a good concordance between the two laboratory techniques in the quantification of *E. coli* abundance. In Denmark, real-time PCR quantification of DNA copies of *L. intracellularis, B. pilosicoli,* and *E. coli* F4 and F18 in fecal samples collected by sock sampling of pen floors [72, 93] is the most commonly performed diagnostic test in cases of intestinal disease in nursery pigs [29]. The proposed cut-offs may be used in the interpretation of real-time PCR results to determine whether *E. coli* F18 is present at high levels. However, it should be noted that our reference variable, the culture-based result, is probably not a gold standard measure of the abundance of *E. coli* in the intestine*.* Furthermore, as we have discussed previously, F18-positive, toxin negative *E. coli* strains are common, and their role as causative agents of PWD is likely minor. Therefore, veterinary practitioners should be cautious when using qPCR based quantification of F18, as this does not necessarily represent the number of ETEC in the sample.

We did not find isolates positive for fimbriae types F5, F6, or F41. This is in line with other European studies that did not detect *E. coli* with these fimbriae [67, 72, 73, 94] or found them at a very low rate [12, 66, 70, 95]. Hence, it is acceptable to focus on F4 and F18 in ETEC diagnostics in PWD cases.

### Fecal pH measurements as a pen-side test

The present study demonstrated that not all cases of PWD are caused by bacteria. Therefore, the use of antimicrobials can be reduced by using a rapid pen-side test, and only administering antimicrobials to cases confirmed to have a bacterial etiology [96]. We hypothesized that the fecal pH would be dependent on underlying pathophysiological mechanisms and possibly have predictive diagnostic capabilities. The foundation for the hypothesis was that ETEC induces secretory diarrhea: the enterotoxins elicit an upregulation of chloride pumps in the intestinal cells, and thus electrolytes and water are pumped into the intestinal lumen [13]. This was expected to alkalize the feces. On the other hand, rotavirus was assumed to cause villus atrophy and thereby induce malabsorptive diarrhea [17], and we expected this pathophysiological process to increase the acidity of the feces [32]. Analogically to the non-infectious causations of PWD [21, 22], one study demonstrated that experimentally induced “gut stress” lowered the fecal pH in broilers [97]. In another study, diarrhea was induced by different mechanisms with certain drugs in 20 humans, and the osmolality and the pH of the feces were measured. They concluded that a fecal pH < 5.3 was suggestive of carbohydrate malabsorptive diarrhea in humans, and pH > 5.6 makes carbohydrate malabsorption an unlikely cause [32]. In the present study, the assumed etiologies, ETEC, rotavirus A, and *S. enterica,* appeared to have a rather small or no association with the fecal pH (Table 11). Therefore, it seemed to be of no avail to estimate the diagnostic capabilities of fecal pH measurements. Nevertheless, rapid pen-side tests are warranted to guide clinical decisions [30]. Recently, a pen-side test based on an enzyme-linked immunosorbent assay (ELISA) was evaluated for post-weaning diarrhea in pigs [96]. This test had acceptable diagnostic sensitivity and a good specificity for the detection of rotavirus. The test had high specificities in detecting *E. coli* F4 and F18; however, the estimated sensitivities were low. The test was validated using real-time PCR as a gold standard reference test, and the authors mentioned that low Ct-values (i.e., high pathogen loads) were associated to a higher probability of testing positive, but a quantitative interpretation was not applied [96]. As previously suggested [1, 11, 79], and according to the results of the present study, it would be relevant to determine the sensitivity of the ELISA-based pen-side test for the detection of high numbers of F4 and F18 *E. coli* shedding.

## Conclusion

The prevalence of diarrhea varied considerably between the nine herds. Watery diarrhea was associated with reduced rectal temperature and alkaline feces. It also appeared to be associated with observably reduced skin elasticity; however, this finding may originate from observer/confirmation bias. The study confirmed ETEC to cause PWD under field conditions, yet cases of PWD where this group of bacteria was not detected in high levels occurred commonly. Diarrhea was associated with shedding of high levels of rotavirus A. Hence, rotaviral enteritis might be a differential diagnosis of PWD, and the clinical significance of this virus should be further investigated. Real-time PCR quantification of more than 17,500 copies of F18 per g of feces indicates the presence of hemolytic *E. coli* F18 at a high level. This is clinically relevant if the *E. coli* strain also has the ability to produce enterotoxins; however, *E. coli* F18 often lacks this ability. The association between microbiological findings in diarrheic pigs and fecal pH was negligible, and hence pH-measurements cannot be used to differentiate between differential diagnoses.

## Supplementary Information

**Additional file 1**: Figures summarizing the sorting of Danish pig herds according to the inclusion criteria.

**Additional file 2**: A protocol for systematic random samplings within a section of pigs.

**Additional file 3**:** Table A**: Some central characteristics of the included herds.

**Additional file 4**: Summary of data from selected European studies: the probability of toxin production given that* E. coli* isolates are F4- or F18-positive.

## Data Availability

The data will be available upon reasonable request to the corresponding author. Data from the present study has previously been presented in preliminary forms [98–100]. Investigations of further objectives from the present study has been reported elsewhere [41].

## Abbreviations

CHR: The Danish central husbandry register
CI: Confidence interval
CT: Cycle threshold
ETEC: Enterotoxigenic *Escherichia coli*
LT: Heat labile toxins
MALDI-TOF: Matrix-assisted laser desorption-ionization time-of-flight
PWD: Post-weaning diarrhea
STa: Heat stabile toxin type A
STb: Heat stabile toxin type B
S. enterica: Salmonella enterica spp. Enterica
