# Supplementary material for: An observational field study of porcine post-weaning diarrhea: clinical and microbiological findings, and fecal pH-measurements as a potential diagnostic tool
Source: Porcine Health Manag. 2023 Jul 11;9:33. doi: 10.1186/s40813-023-00325-x (PMC10334583; doi:10.1186/s40813-023-00325-x)
Supplement: Supplementary file 1 — Additional file 1: Figures summarizing the sorting of Danish pig herds according to the inclusion criteria. [file 40813_2023_325_MOESM1_ESM.pdf]

Supplementary File A

Sampling stage 1

The objective of sampling stage 1 was to identify herds accommodating pigs meeting inclusion criteria A, B, C, and D. Such pigs could be found in herds meeting the inclusion criteria for herds.

In Denmark, prescriptions and purchases of all veterinary medical products must be registered in VetStat [1] and all pig herds are registered in the Danish Central Husbandry Register (CHR) [2]. Data on purchases of medicinal zinc oxide products for two periods of three to four months was obtained from VetStat and combined with CHR data on the number of pigs in each age group and postal code. Hereby, 216 herds suspected to meet inclusion criteria 1, 2, 5, and 6 were revealed, as illustrated in Figure A (see below). There were not enough herds identified in this first sampling round in April and May 2019. This was the reason for performing an additional sampling round (see Figure A, left arm) using data on zinc oxide purchase from another period and changing the fifth inclusion criterion from 2 hours to 2.5 hours of driving range from the University of Copenhagen, Frederiksberg (Textbox 2). The next step was to establish whether these herds actually met the inclusion criteria (Textbox 2), and for this purpose, we conducted short telephone interviews in April and May, and in August, September, and October 2019. The sorting is summarized in Figure B (see below). Most of the herds (n = 212) could be categorized through short telephone interviews with the herd owner or trusted personnel (n=179) or by a further look into register data (n = 33). If herds met all the inclusion criteria (n = 10), they were given the opportunity to participate in the study.

Sampling stage 2

The two objectives of the second stage were to characterize the PWD outbreaks clinically, including the prevalence of diarrhea, and to obtain a patient database from which cases and controls could be selected in sampling stage 3. Herds included in the first sampling stage were visited on a day when they reported an outbreak of diarrhea deemed to require antimicrobial batch medication for more than 100 animals. Outbreaks were only examined if they occurred on a day when an investigation team was on-call, and sample processing was possible the following day. The team was on-call for 16 days in the period May 8 to June 202019, and four out of nine reported outbreaks were examined in this period. From August and on, the team scheduled the days on-call based on high risk periods in the herds that had not yet been visited. For example, Herd E, K, and M usually experienced diarrhea three to four days after weaning, and thus the team scheduled days corresponding to two to five days after planned weaning. The single eligible herd that was not visited (Figure B, see below) was identified and enrolled late in the study period, weaned batches at a five week rate, and the PWD outbreaks occurred irregularly. Therefore, the herd only reported one PWD outbreak before the data collection was terminated, and our team was not on call this day.

The herd visits were performed from May to October 2019 (see Supplementary Table A for exact dates). A sample of 90 to 113 pigs was selected for a cross-sectional investigation of clinical signs in each herd. Pigs were selected by systematic random sampling (see Supplementary File B for a comprehensive description of the procedure) among all the newly weaned pigs assigned to oral batch medication against PWD on the given day. All selected pigs were subjected to a clinical examination, fecal measurements, and an ID-number was written on their dorsum, so they could be identified if they were selected in sampling stage 3.

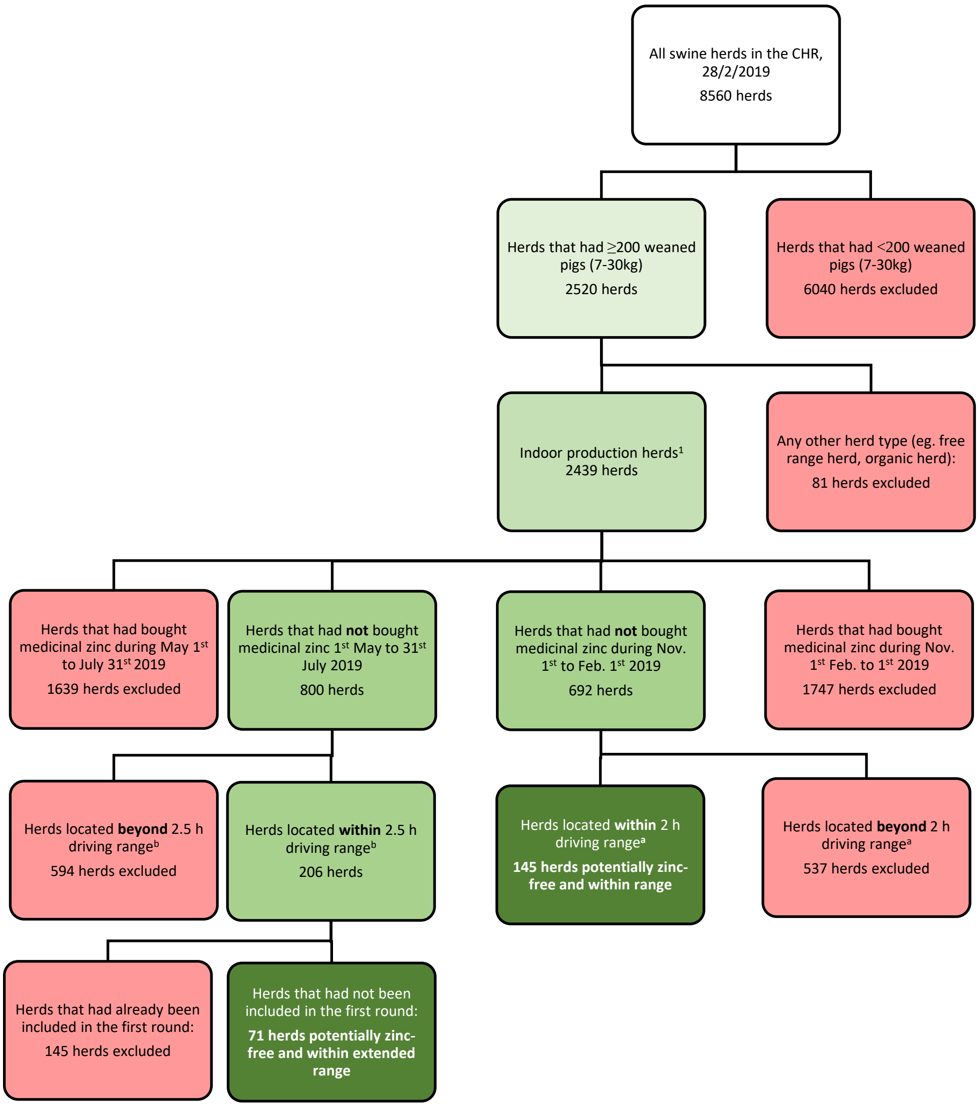

Figure A: Sampling of Danish pig herds suspected not to use medicinal zinc oxide for their newly weaned pigs not based on register data.

The arm to the left represents a second (additional) sampling round conducted in the autumn of 2019. Here, the purchase of medicinal zinc was based on updated records from VetStat, and the geographical range was extended.

[a]: Including the islands Amager, Funen, Lolland, Falster, Tåsinge, and Zealand (see Supplementary Figure A).

[b]: Including the islands Amager, Funen, Lolland, Falster, Tåsinge, and Zealand and additionally the eastern part of the Triangle Region in Jutland (see Supplementary Figure A).

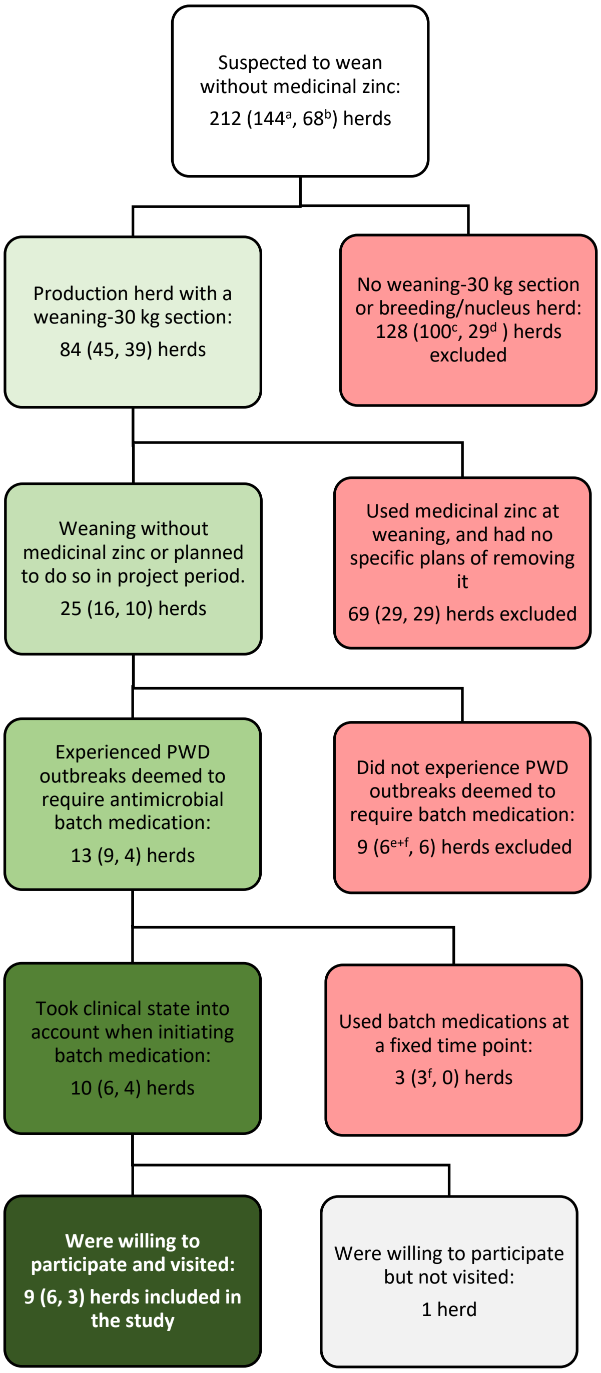

Figure B: How 212 herds met the inclusion criteria in an observational field study of post-weaning diarrhea (PWD).

The numbers specified in parentheses refer to the sorting from the first and second sampling rounds, respectively.

[a]: There were 145 shortlisted herds; however, one could not be classified.
[b]: There were 71 shortlisted herds; however, three could not be classified.
[c]: Including five breeding/nucleus herds.
[d]: Including seven breeding/nucleus herds.
[e]: Two of these herds were given the opportunity to participate, although they reported that PWD outbreaks were rare. They did not report any PWD outbreaks during the study period.
[f]: Two herds were categorized as not experiencing diarrhea. However, they did use routine antimicrobial batch treatments on day seven post-weaning due to respiratory disease. It cannot be ruled out that this treatment also prevented post-weaning diarrhea on days 7 to 14 post weaning.

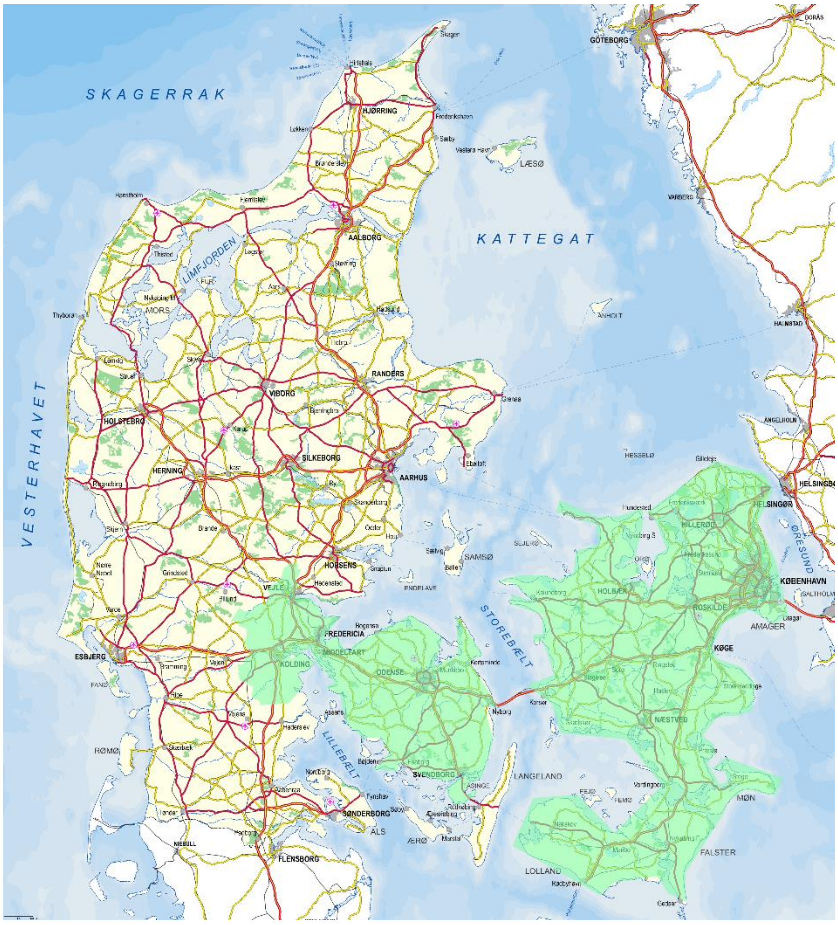

Figure C: Illustration of the geographical area in Eastern denmark (colored green) from which zinc-free herds were recruited in the study of post-weaning diarrhea.

Map edited and downloaded from The Agency for Data Supply and Efficiency [3].

References

1. Stege H, Bager F, Jacobsen E, Thougaard A. VETSTAT - the Danish system for surveillance of the veterinary use of drugs for production animals. Prev Vet Med. 2003;57:105–15.

2. Birkegård AC, Fertner ME, Jensen VF, Boklund A, Toft N, Halasa T, et al. Building the foundation for veterinary register-based epidemiology: A systematic approach to data quality assessment and validation. Zoonoses Public Health. 2018;65:936–46.

3. The Agency for Data Supply and Efficiency. Map Viewer [Internet]. [cited 2022 Aug 16]. Available from: http://eng.sdfe.dk/datadistribution/map-viewer
