# Supplementary material for: An observational field study of porcine post-weaning diarrhea: clinical and microbiological findings, and fecal pH-measurements as a potential diagnostic tool
Source: Porcine Health Manag. 2023 Jul 11;9:33. doi: 10.1186/s40813-023-00325-x (PMC10334583; doi:10.1186/s40813-023-00325-x)
Supplement: Supplementary file 2 — Additional file 2: A protocol for systematic random samplings within a section of pigs. [file 40813_2023_325_MOESM2_ESM.pdf]

Systematic random sampling of pigs

Objective

To select a systematic random sample from a group (e.g. a herd, a section, or a pen) of pigs.

Materials

- Marker spray in two different colors (e.g., red and blue)
- Dice (or any other mean to randomly select a fraction)
- Pig board (optional)

Method

Table 1 outlines the method in eight bullets. An example is provided in the right side of the table.

Table 1: Eight step guide to systematic random sampling of pigs with example

<table>
<tr><th></th><th>Method</th><th>Example</th></tr>
<tr><td>1</td><td>Decide on the least satisfactory sample size (SS) based on a sample size calculation.</td><td>The least satisfactory sample size is 165 pigs.</td></tr>
<tr><td>2</td><td>Determine the approximate number of animals in the source population (SP).</td><td>The section that makes up the source population accommodates ~440 pigs.</td></tr>
<tr><td>3</td><td>Calculate a SS/SP-ratio: sample size/source population size.</td><td>SS/SP-ratio = 165/440 = 0.375</td></tr>
<tr><td>4</td><td>Round up the SS/SP-ratio to the nearest possible value in the Table 2 (see below). Pick the fraction matching the SS/SP-ratio.</td><td>0.375 is rounded up to 0.4. I.e., the fraction 2/5 is picked.</td></tr>
<tr><td>5</td><td>Calculate: Fraction*SP. Is this sample size too large to be feasible to use? If yes, consider a smaller fraction.</td><td>Fraction*SP = 0.4*440 = 176. This sample size is okay to handle. It is above 165 and not too large.</td></tr>
<tr><td>6</td><td>When a fraction has been picked, determine the denominator of the fraction. Based on the denominator, select a set of two to six symbols, as outlined in Table 3 below.</td><td>The denominator of the fraction is 5, so this set of symbols is picked:
-   | ●   - |</td></tr>
<tr><td>7</td><td>Mark all pigs in the source population with the selected symbols using two marker sprays of different colors. The nearest pig will be given the first symbol on the dorsum, the second pig will be given the second symbol and so forth. The order of symbols is repeated. Always use the same hand to paint (e.g., only your right hand) and say the symbols aloud to keep track!</td><td>The symbols are repeatedly painted on the dorsum of the pigs.
Hint: Using a pig board, an assistant can direct all pigs into one end of a pen. Pigs are encouraged to run to the other side of the pen one at a time, and pass by the person marking them.</td></tr>
<tr><td>8</td><td>Now the source population has been systematically divided into equally sized groups marked with symbols. The numerator of the picked fraction defines how many groups must be included in the sample. A dice is rolled to select group(s) characterized by symbol(s) according to Table 3 seen below. www.random.org or a lottery can be used instead of a dice.</td><td>A dice is rolled until two out of the five fractions have been picked to be included in the sample:
⚁ All pigs with | are included
⚅ Not in the used system, roll again…
⚁ Already included, roll again…
⚃ All pigs with - are included</td></tr>
</table>

Table 2: Sample size/source population ratio and corresponding fraction

SS/SP ratio	Fraction
1	1/1
0.83	5/6
0.8	4/5
0.75	3/4
0.66	2/3
0.6	3/5
0.5	1/2
0.4	2/5
0.33	1/3
0.25	1/4
0.2	1/5
0.13	1/6

Table 3: What systematic random sample to include according to dice values and denominator

**Dice value/ Denominator**	⚀	⚁	⚂	⚃	⚄	⚅
**2**	●	●				
**3**	-	│	●			
**4**	-	│	-	│		
**5**	-	│	●	-	│	
**6**	-	│	●	-	│	●
