# Supplementary material for: An observational field study of porcine post-weaning diarrhea: clinical and microbiological findings, and fecal pH-measurements as a potential diagnostic tool
Source: Porcine Health Manag. 2023 Jul 11;9:33. doi: 10.1186/s40813-023-00325-x (PMC10334583; doi:10.1186/s40813-023-00325-x)
Supplement: Supplementary file 3 — Additional file 3: Table A: Some central characteristics of the included herds. [file 40813_2023_325_MOESM3_ESM.pdf]

Supplementary table A: Description of Danish herds weaning without medicinal zinc oxide included in a study of post-weaning diarrhea

Herd	Geography	Date of insertion[a]	Date of visit[a]	Days after insertion	Antimicrobial used	Batch size	Herd type	SPF status
A	Zealand	May 9	May 13	4	Lincomycin-spectinomycin	~ 320	W-30	SPF + Myc + Ap6 + Ap12
B	Zealand	May 23[b]	May 27	4	Tylosin	~ 139	F-L	Non-SPF
C	Zealand	May 24	May 28	4	Lincomycin-spectinomycin	~ 600	W-F	Non-SPF
D	Funen	June 7	June 11	4	Lincomycin-spectinomycin	~ 500	W-30	SPF+ Myc + Ap6 + Ap12
E	Funen	Aug. 19	Aug. 22	3	Tylosin	417	W-30	SPF + Ap6 + Ap12
F	Funen	Aug. 19	Aug. 28	8	Tylosin	250	W-F	SPF+ Ap6+ Ap12
G	Triangle region	Oct. 7.	Oct. 9	2	Neomycin	1135	W-30	SPF + Myc
K	Funen	Oct 23	Oct. 27	4	Doxycyclinhyclat	546	W-30	SPF + Myc
M	Funen	Oct 25	Oct. 29	4	Doxycyclinhyclat	432	B-30	SPF + Myc + Ap6 + Ap12 + sanPRRS1 + sanPRRS2

[a]: All dates were in 2019

[b]: Two litters weaned from nursing sows were added to the batch on the day of the herd visit.

W-30: Production from weaning to 30 kg

F-L: Full-line production

W-F: Production from weaning to finish

B-30: birth to 30 kg production

SPF: Declared free of *Mycoplasma hyopneumoniae, Actinobacillus pleuropneumoniae*, porcine respiratory and reproductive syndrome virus types 1 and 2, *Brachyspira hyodysenteriae*, toxin-producing *Pasteurella multocida*, Sarcoptes scabiei var. suis, and Haematopinus suis.

Myc: infected with *Mycoplasma hyopneumoniae*

Ap6/Ap12: infected with *Actinobacillus pleuropneumoniae* type 6/type 12.

sanPRRS1 and sanPRRS2: Eradication of PRRS type 1 and type 2 in progress.
