# Supplementary material for: An observational field study of porcine post-weaning diarrhea: clinical and microbiological findings, and fecal pH-measurements as a potential diagnostic tool
Source: Porcine Health Manag. 2023 Jul 11;9:33. doi: 10.1186/s40813-023-00325-x (PMC10334583; doi:10.1186/s40813-023-00325-x)
Supplement: Supplementary file 4 — Additional file 4: Summary of data from selected European studies: the probability of toxin production given that E. coli isolates are F4- or F18-positive. [file 40813_2023_325_MOESM4_ESM.pdf]

Table B: The fraction (n/N) of *E. coli* isolates from weaned pigs with Fimbria type F4 or F18 carrying genes encoding enterotoxins (Toxins) in the present and other European studies.

Reference	Country	Source	Year	Age	N Herds	Zinc usage	Toxins/ F4	Toxins/ F18
Present study	Denmark	Survey	2019	0-14 days post-weaning	9	No	6/7	45/80
Eriksen et al., 2021	Denmark	Survey	2019	0-14 days post-weaning	2	No	3/3	164/282
Weber and Haugegaard, 2020[a]	Denmark	Diagnostic submissions	2019	Nursery pigs of all ages	Unknown	Most likely[b]	43/43	46/96
Morsing et al., in review	Denmark	Survey	2018	0-14 days post-weaning	10	No	9/9	32/42
García et al., 2020	Denmark	Diagnostic submissions,	2018[c]	Nursery pigs of unknown age	30	Most likely[b]	34/34	16/20
Weber et al., 2017	Denmark	Survey	2014	14-28 days post-weaning	3	Most likely[b]	0	41/46
Luppi et al., 2016	Belgium, France, Germany, Italy, The Netherlands	Diagnostic submissions	2012-2014	1-3 weeks post-weaning	280	Not specified	116/153	61/115
Zajacova et al., 2012	Czech republic	Diagnostic submissions	2005-2009	25% pre-weaning piglets, 75% post-weaning pigs	Not specified	Not specified	183/183	64/103[d]
Vu Khac et al., 2006	Slovakia	Survey	2001-2003	Nursery pigs of unknown age.	20	Not specified	18/18	22/35
Frydendahl, 2002	Denmark	Diagnostic submissions	1999-2000	4–8-week-old pigs		Not specified	97/97	64/86
Osek, 2000	Poland	Survey	Not specified	4-6 weeks old weaned pigs	8	Not specified	0	4/10
Osek et al., 1999	Poland	Unclear	Not specified	Nursery pigs of unknown age	Not specified	Not specified	9/9	21/29
Osek, 1999	Poland	Survey	1995-1997	4-6 weeks old weaned pigs	18	Not specified	71/71	10/11

Some of the studies (Frydendahl, 2002; Osek et al., 1999; Weber and Haugegaard, 2020; Zajacova et al., 2012) included some diagnostic submissions from pigs suffering from edema disease. Some of the *E. coli* isolates carried genes encoding F18 and verotoxins but not enterotoxins. The association between F18 and enterotoxins would likely be stronger in these studies, if only PWD cases had been included.

[a]: We were able to merely include the isolates from weaned pigs after correspondence with the authors.

[b]: In-feed medicinal zinc has been used in almost all Danish indoor productions from 2007 and onwards until at least 2019 (DANMAP, 2018; Nielsen et al., 2021).

[c]: We have only included the isolates from 2018, as the F4 isolates from 2019 originated from the diagnostic laboratory reporting results in the report by Weber and Haugegaard (2020), and the 20 F18-positive isolates stem from the present study (Herds A, B, C, D and E). The seven older isolates (1989-1992 & 1970s) was considered irrelevant.

[d]:This study did not test for STb. Thus, some F18-positive but apparently toxin-negative isolates might have been positive for STb.

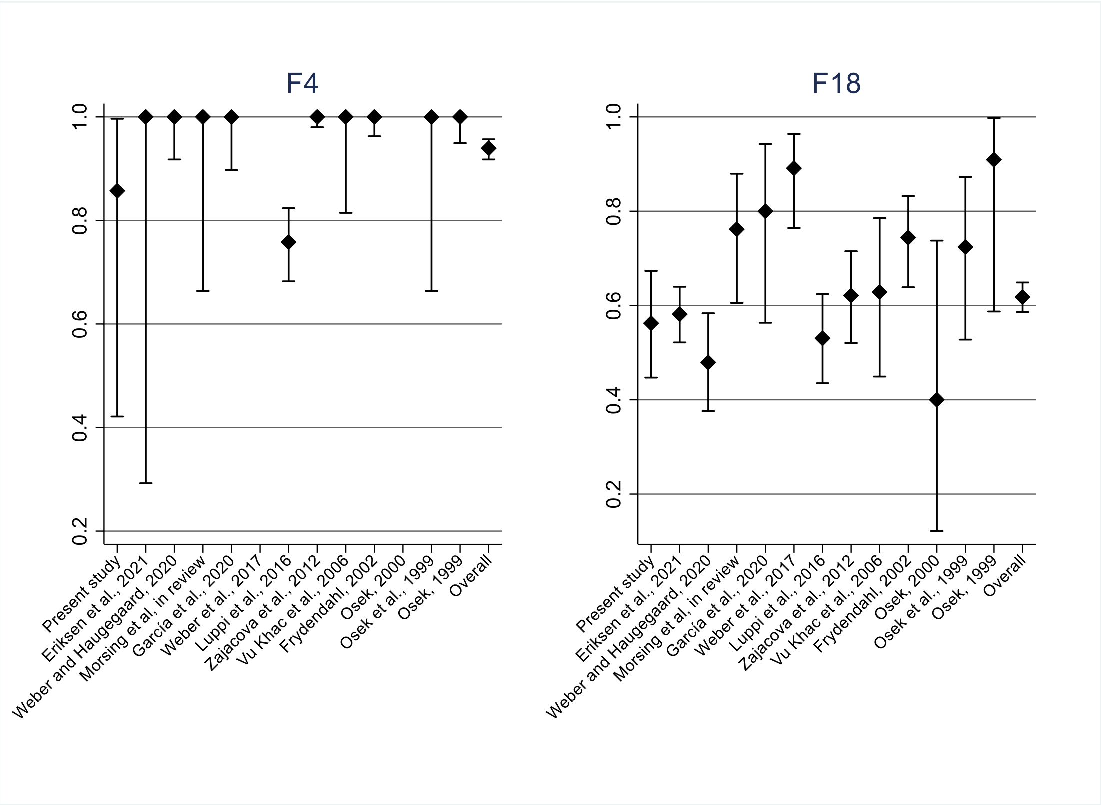

Figure D: Estimated probabilities (proportions with 95% Clopper-Pearson confidence intervals) of *E. coli* isolates with F4 or F18 carrying at least one enterotoxin in in 13 European studies.

Further details of the studies are specified in supplementary Table B (see above).

References

DANMAP, 2018. DANMAP 2017 - Use of antimicrobial agents and occurrence of antimicrobial resistance in bacteria from food animals, food and humans in Denmark.

Eriksen, E.Ø., Kudirkiene, E., Christensen, A.E., Agerlin, M.V., Weber, N.R., Nødtvedt, A., Nielsen, J.P., Hartmann, K.T., Skade, L., Larsen, L.E., Pankoke, K., Olsen, J.E., Jensen, H.E., Pedersen, K.S., 2021. Post-weaning diarrhea in pigs weaned without medicinal zinc: risk factors, pathogen dynamics, and association to growth rate. Porc Health Manag 7, 1–19. https://doi.org/10.1186/s40813-021-00232-z

Frydendahl, K., 2002. Prevalence of serogroups and virulence genes in Escherichia coli associated with postweaning diarrhoea and edema disease in pigs and a comparison of diagnostic approaches. Veterinary Microbiology 85, 169–182. https://doi.org/10.1016/S0378-1135(01)00504-1

García, V., Gambino, M., Pedersen, K., Haugegaard, S., Olsen, J.E., Herrero-Fresno, A., 2020. F4- and F18-Positive Enterotoxigenic Escherichia coli Isolates from Diarrhea of Postweaning Pigs: Genomic Characterization. Appl. Environ. Microbiol. 86. https://doi.org/10.1128/AEM.01913-20

Luppi, A., Gibellini, M., Gin, T., Vangroenweghe, F., Vandenbroucke, V., Bauerfeind, R., Bonilauri, P., Labarque, G., Hidalgo, Á., 2016. Prevalence of virulence factors in enterotoxigenic

Escherichia coli isolated from pigs with post-weaning diarrhoea in Europe. Porc Health Manag 2, 1–6. https://doi.org/10.1186/s40813-016-0039-9

Morsing, M.K., Larsen, I., Pedersen, K.S., Weber, N.R., Nielsen, J.P., 2022. The Prevalence of Post-Weaning Diarrhoea and Role of Enterotoxigenic Escherichia Coli in Ten Danish Nursery Pig Herds Not Using Medicinal Zinc Oxide in the Feed. https://doi.org/10.2139/ssrn.4090430

Nielsen, C.L., Kongsted, H., Sørensen, J.T., Krogh, M.A., 2021. Antibiotic and medical zinc oxide usage in Danish conventional and welfare-label pig herds in 2016–2018. Preventive Veterinary Medicine 189, 105283. https://doi.org/10.1016/j.prevetmed.2021.105283

Osek, J., 2000. Virulence factors and genetic relatedness of Escherichia coli strains isolated from pigs with post-weaning diarrhea. Veterinary Microbiology 71, 211–222. https://doi.org/10.1016/S0378-1135(99)00168-6

Osek, J., 1999. Prevalence of virulence factors of Escherichia coli strains isolated from diarrheic and healthy piglets after weaning. Vet Microbiol 68, 209–217. https://doi.org/10.1016/s0378-1135(99)00109-1

Osek, J., Gallien, P., Truszczyñski, M., Protz, D., 1999. The use of polymerase chain reaction for determination of virulence factors of Escherichia coli strains isolated from pigs in Poland. Comparative Immunology, Microbiology and Infectious Diseases 22, 163–174. https://doi.org/10.1016/S0147-9571(98)00083-6

Vu Khac, H., Holoda, E., Pilipcinec, E., Blanco, M., Blanco, J.E., Mora, A., Dahbi, G., López, C., González, E.A., Blanco, J., 2006. Serotypes, virulence genes, and PFGE profiles of Escherichia coliisolated from pigs with postweaning diarrhoea in Slovakia. BMC Vet Res 2, 10. https://doi.org/10.1186/1746-6148-2-10

Weber, N., Haugegaard, S., 2020. Forekomst af virulensfaktorer i hæmolytiske E.coli-bakterier isoleret fra pattegrise og smågrise (No. 1198).

Weber, N.R., Nielsen, J.P., Hjulsager, C.K., Jorsal, S.E., Haugegaard, S., Hansen, C.F., Pedersen, K.S., 2017. Comparison of bacterial culture and qPCR testing of rectal and pen floor samples as diagnostic approaches to detect enterotoxic Escherichia coli in nursery pigs. Preventive Veterinary Medicine 143, 61–67. https://doi.org/10.1016/j.prevetmed.2017.05.009

Zajacova, Z.S., Konstantinova, L., Alexa, P., 2012. Detection of virulence factors of Escherichia coli focused on prevalence of EAST1 toxin in stool of diarrheic and non-diarrheic piglets and presence of adhesion involving virulence factors in astA positive strains. Veterinary Microbiology 154, 369–375. https://doi.org/10.1016/j.vetmic.2011.07.029
